# Alterations in working memory maintenance of fearful face distractors in depressed participants: An ERP study

**DOI:** 10.1167/jov.23.1.10

**Published:** 2023-01-18

**Authors:** Chaoxiong Ye, Qianru Xu, Xueqiao Li, Elisa Vuoriainen, Qiang Liu, Piia Astikainen

**Affiliations:** https://orcid.org/0000-0002-8301-7582; https://orcid.org/0000-0003-1579-6972; https://orcid.org/0000-0003-4842-7460; 1 Institute of Brain and Psychological Sciences, Sichuan Normal University, Chengdu, China; 2 Department of Psychology, University of Jyväskylä, Jyväskylä, Finland; 3 Center for Machine Vision and Signal Analysis, University of Oulu, Oulu, Finland; 4 Faculty of Social Sciences, Tampere University, Tampere, Finland; 5 Center for Machine Vision and Signal Analysis, University of Oulu, Oulu, Finland; 6 Department of Psychology, University of Jyväskylä, Jyväskylä, Finland; 7 Faculty of Social Sciences, Tampere University, Tampere, Finland; 8 Institute of Brain and Psychological Sciences, Sichuan Normal University, Chengdu, China; 9 Department of Psychology, University of Jyväskylä, Jyväskylä, Finland

**Keywords:** contralateral delay activity, depression, ERP, face distractor, negative expression, visual working memory

## Abstract

Task-irrelevant threatening faces (e.g., fearful) are difficult to filter from visual working memory (VWM), but the difficulty in filtering non-threatening negative faces (e.g., sad) is not known. Depressive symptoms could also potentially affect the ability to filter different emotional faces. We tested the filtering of task-irrelevant sad and fearful faces by depressed and control participants performing a color-change detection task. The VWM storage of distractors was indicated by contralateral delay activity, a specific event-related potential index for the number of objects stored in VWM during the maintenance phase. The control group did not store sad face distractors, but they automatically stored fearful face distractors, suggesting that threatening faces are specifically difficult to filter from VWM in non-depressed individuals. By contrast, depressed participants showed no additional consumption of VWM resources for either the distractor condition or the non-distractor condition, possibly suggesting that neither fearful nor sad face distractors were maintained in VWM. Our control group results confirm previous findings of a threat-related filtering difficulty in the normal population while also suggesting that task-irrelevant non-threatening negative faces do not automatically load into VWM. The novel finding of the lack of negative distractors within VWM storage in participants with depressive symptoms may reflect a decreased overall responsiveness to negative facial stimuli. Future studies should investigate the mechanisms underlying distractor filtering in depressed populations.

## Introduction

Visual working memory (VWM) is a fundamental cognitive system that provides an online workspace for effectively accessing and updating of information when a visual stimulus disappears (Luck & Vogel, 1997; Luck & Vogel, 2013). VWM supports some of the most essential aspects of higher-level cognition (Johnson et al., 2013), including fluid intelligence and attention control (Fukuda, Vogel, Mayr, & Awh, 2010; Unsworth, Fukuda, Awh, & Vogel, 2014). However, the capacity of VWM is extremely limited (Fukuda, Awh, & Vogel, 2010; Ma, Husain, & Bays, 2014; Vogel & Awh, 2008), and the visual system often encounters task requirements that exceed the limits of VWM. Selective regulation of access to task-relevant stimuli in VWM is critical, as is filtering for task-irrelevant distractors; therefore, a considerable amount of literature has appeared on the topic of distractor filtering in VWM (Allon & Luria, 2019; Feldmann-Wustefeld & Vogel, 2019; Hakim, Feldmann-Wüstefeld, Awh, & Vogel, 2020; Lorenc, Mallett, & Lewis-Peacock, 2021; McNab & Dolan, 2014; Song, Chang, & Zhou, 2021; Vogel, McCollough, & Machizawa, 2005).

In humans, human faces are biologically and socially significant stimuli (Langton, Law, Burton, & Schweinberger, 2008; Ro, Russell, & Lavie, 2001). Although humans are experts in face processing (Gauthier, Skudlarski, Gore, & Anderson, 2000), face distractors can interrupt an ongoing VWM task and can be difficult to filter (Gambarota & Sessa, 2019; Stout, Shackman, & Larson, 2013). Previous studies using behavioral, event-related potential (ERP), and functional magnetic resonance imaging techniques have found that participants experience difficulties when attempting to filter fearful face distractors from VWM (Stout et al., 2013; Stout, Shackman, Johnson, & Larson, 2015; Stout, Shackman, Pedersen, Miskovich, & Larson, 2017). In everyday life, for example, this might be observed when a person is reading news on the internet and an emotional face in a pop-up advertisement makes the person forget what was just read.

Researchers have investigated face storage (Meconi, Luria, & Sessa, 2014; Sessa & Dalmaso, 2016; Sessa, Luria, Gotler, Jolicoeur, & Dell'Acqua, 2011; Sessa, Schiano Lomoriello, & Luria, 2018; Sessa, Tomelleri, Luria, Castelli, Reynolds, & Dell'Acqua, 2012) and the ability to filter emotional face distractors in VWM (Salahub & Emrich, 2020; Stout et al., 2013; Ye, Xu, Liu, Cong, Saariluoma, Ristaniemi, & Astikainen, 2018; Zhang, Ye, Roberson, Zhao, Xue, & Liu, 2021) using an ERP component referred to as contralateral delay activity (CDA, also known as sustained posterior contralateral negativity) (Sessa et al., 2011; Sessa et al., 2012). The CDA component is widely used as an ERP marker of the visual information load stored in VWM (Adam, Robison, & Vogel, 2018; Feldmann-Wustefeld & Vogel, 2019; Feldmann-Wustefeld, Vogel, & Awh, 2018). An increase in the number of representations in VWM leads to a larger CDA amplitude (Luck & Vogel, 2013; Luria, Balaban, Awh, & Vogel, 2016; Vogel & Machizawa, 2004). Therefore, the CDA amplitude reveals the VWM resources allocated to representations.

Stout et al. (2013) developed a face-filtering task to investigate face-distractor filtering in VWM using the CDA component. They instructed participants to memorize the identity of a neutral target face and to ignore a distractor face (a neutral or a fearful face) (Stout et al., 2013). Their results showed that the CDA amplitude did not differ between the trials that had a neutral face as the distractor and the trials that had no distractor, suggesting that the participants did not store neutral face distractors in VWM. However, the CDA amplitude was larger for trials with fearful distractors than for trials with no distractors, suggesting that the participants failed to filter out fearful face distractors and automatically stored them in VWM.

A recent study by Salahub and Emrich (2020), who used an ERP component called N2pc as an index of attention selection (Eimer, 1996; Luck & Hillyard, 1994a; Luck & Hillyard, 1994b) and CDA as the VWM maintenance indicator, found that fearful face distractors elicited an increase in the N2pc amplitude and a relative increase in the CDA amplitude in participants. This pattern of results suggests that increased attention to a fearful distractor also increases the likelihood that the face will be held in VWM. We recently used a face-filtering task to show that participants with a high VWM capacity could filter out all distractors (happy, neutral, and angry faces), whereas participants with a low VWM showed effective filtering activity only for happy faces (Ye et al., 2018). We interpreted this result as indicating that individuals with limited VWM capacity have particular difficulty filtering potentially threatening distractors. A similar study by Zhang et al. (2021) showed that participants in a personal relative deprivation group (i.e., individuals who felt more deprived compared to the referent level) had difficulties filtering neutral and angry face distractors, but they were able to filter out happy face distractors. These findings are in agreement with those observed by Ye et al. (2018) for participants with low VWM capacity. In summary, previous studies have used the CDA component to investigate whether participants can filter threatening negative faces (i.e., fearful and angry faces), neutral faces, and positive faces (i.e., happy faces) as distractors of VWM. However, the ability to filter non-threatening negative face (e.g., sad face) distractors from VWM has not been systematically studied.

The ability to filter sad faces from VWM is an interesting and important aspect to address in both the healthy population and the population with mood disorders. Previous studies have suggested that the presence of a processing bias for sad faces in participants with depressive symptoms. Behavioral studies and brain activity measurements of facial expression processing have demonstrated pre-attentive perceptual (Ruohonen, Alhainen, & Astikainen, 2020; Xu et al., 2018; Zhang, He, Chen, & Wei, 2016; Zhao et al., 2015), attentive (Dai & Feng, 2012), and VWM (Linden, Jackson, Subramanian, Healy, & Linden, 2011) biases toward sad faces in patients with preclinical and clinical depression (for a review, see Gotlib & Joormann, 2010). These empirical findings are in agreement with Beck's cognitive theory of depression, which states that the negative schemas of depressed individuals skew their information processing toward negative information (Beck, 1967; Beck, 2008); indeed, previous findings show that the storage of sad faces is enhanced in depression (Linden et al., 2011). To date, these studies have only applied CDA to investigate the filtering of non-face objects in depression (e.g., Owens, Koster, & Derakshan, 2012); consequently, the effect of depressive symptoms on the ability to filter sad face distractors from VWM is unknown. To the best of our knowledge, no previous studies have used the CDA component to investigate the ability to filter sad face distractors in VWM.

Many investigations of the filtering mechanism in VWM for face distractors have used face stimuli as both targets and distractors in healthy or anxious participants (Salahub & Emrich, 2020; Stout et al., 2013; Stout et al., 2015; Stout et al., 2017; Ye et al., 2018; Zhang et al., 2021). Some researchers have used different colored frames surrounding the face to help the participants distinguish target from distractor stimuli (e.g., to remember faces with a red frame and ignore faces with a yellow frame) (Stout et al., 2013). Therefore, when participants need to filter the distractors, they must first correctly use the color frames to distinguish whether they are viewing a target or a distractor, and then they must remember the targets and filter the distractors. This leads to a potential problem, as the participants may fail to use the color frames to distinguish the targets from the distractors. This would then cause a failure in the face-filtering task even before the VWM maintenance phase. Thus, the filtering failure in the previous classical filtering paradigm (using different colored frames surrounding the face to distinguish the targets and distractors) may be caused by other early cognitive processes rather than the VWM process.

In the present study, we used a novel filtering paradigm to compare the ability to filter negative emotional faces from VWM between participants with depressive symptoms and non-depressed participants. We applied a task consisting of targets and distractors of different categories (i.e., the targets were colored squares and the distractors were faces). We anticipated that this approach would reduce the contribution of target selection and related attentional control in the task and would therefore reflect mostly the storage of objects. The aim of our paradigm was to circumvent the difficulty encountered in target selection in previous studies that used faces as both targets and distractors (Salahub & Emrich, 2020; Stout et al., 2015; Stout et al., 2013; Stout et al., 2017; Ye et al., 2018; Zhang et al., 2021). Here, the CDA component was measured while the participants conducted a filtering task. Comparison of the CDA amplitude under distractor conditions with that of the baseline condition, which did not include any distractors, allowed us to investigate whether the depressed and non-depressed participants were equally able to filter fearful and sad face distractors.

As in some previous VWM studies (Owens et al., 2012; Owens, Koster, & Derakshan, 2013), we enrolled participants with an increased number of depressive symptoms (depressed group) and participants with no/few depressive symptoms (control group). We expected that participants in the control group would have difficulty filtering out fearful face distractors from VWM, as shown by previous studies (Salahub & Emrich, 2020; Stout et al., 2013), possibly because threat perception is prioritized in human information processing for evolutionary reasons (LeDoux, 1996). The pattern of results was expected to be similar in the depressed and control groups regarding the ability to filter fearful face distractors, because the attentive bias in depression is not evident for threat contents (Armstrong & Olatunji, 2012). The control group was expected to show a more efficient ability to filter sad faces than fearful faces, because sad faces do not pose any threat and are therefore probably not as attention capturing as fearful faces. In the depressed group, we expected that the negative attentive bias in depression would cause difficulty in suppressing attention to sad distractors, thereby leading to a failure to filter sad face distractors from VWM.

## Methods

### Participants

All participants in the two groups (depressed group and control group) were recruited via email lists, advertisement flyers distributed around the Jyväskylä area, and notice board announcements at the University of Jyväskylä. All participants provided written informed consent before participating in the experiment. The procedures of the study complied with the tenets of the Declaration of Helsinki and were approved by the ethical committee of Central Finland Central Hospital.

Adequate power for the comparison at the group level was ensured by a priori determination of the sample size by a power analysis based on the predicted effect size using G∗Power 3.1.9.2 (Faul, Erdfelder, Lang, & Buchner, 2007). Previous studies on the CDA component have shown a medium or large effect size on the manipulation of filtering conditions (Owens et al., 2013; Stout et al., 2013; Ye et al., 2018). Thus, we predicted a medium effect size (η*_p_*^2^ = 0.06) for our experimental design. With a statistical power of (1 – β) = 0.90 and a significance level of 0.05, the suggested total sample size was approximately 36 participants (18 participants in the depressed group and 18 participants in the control group).

As in the study by Owens et al. (2012), we recruited two groups of participants in the present study. In total, 48 Finnish-speaking participants were first recruited for the two groups in this study. Twelve participants (25%) were excluded because of extensive electroencephalogram (EEG) artifacts and eye movements. The proportion of excluded participants in this study was similar to the proportions reported in previous facial VWM studies using CDA (e.g., 36% participants in the study by Sessa et al., 2011; 29% participants in the study by Stout et al., 2013; 24% participants in the study by Ye et al., 2018). The results reported here are therefore based on data from the remaining 36 participants: 18 in the depressed group (30.94 ± 7.06 years old; seven males, 11 females) and 18 in the control group (26.61 ± 6.29 years old; four males, 14 females). The inclusion criteria were a score of nine or less on the Beck Depression Inventory II (BDI-II) (Beck, Steer, & Brown, 1996) for the control group and a score of 14 or higher on the BDI-II for the depressed group.

The inclusion criteria for all participants were normal or corrected-to-normal vision, normal color vision, right-handedness, and age between 18 and 40 years. The exclusion criteria for all participants were self-reports of brain damage, current substance abuse, or neurological disorders (except migraines [not recently active] or fibromyalgia). Additional exclusion criteria for the depressed group were current or previous severe psychiatric disorders and symptoms other than depression and anxiety symptoms. Additional exclusion criteria for the control group were current or previous diagnosis of depression, any other psychiatric diagnosis, and current use of medication that could affect the central nervous system.

Anxiety symptoms were assessed in all participants by having each fill out a questionnaire on anxiety symptoms. The anxiety symptoms of all participants were measured using the anxiety subscale of the Depression Anxiety Stress Scale–Anxiety (DASS-A) (Lovibond & Lovibond, 1996).

The mean BDI-II scores were 26.83 (*SD* = 6.76; range, 17–42) in the depressed group and 2.56 (*SD* = 1.79; range, 0–5) in the control group. The mean DASS-A scores were 8 (*SD* = 4.17; range, 1–15) in the depressed group and 1.83 (*SD* = 2.77; range, 0–10) in the control group. Eleven of the depressed group participants had been diagnosed with depression (six participants had a diagnosis given within 1 year of the study, and five participants had a diagnosis given more than a year before the study); the remaining seven participants had no definitive diagnosis. Four participants in the depressed group reported having an additional diagnosis of an anxiety disorder, and one reported having an anankastic personality disorder.

Both the BDI-II scores and DASS-A scores were significantly higher for the participants in the depression group than in the control group, *t*(34) = 14.733, *p* < 0.001, Cohen's *d* = 4.910, BF_10_ > 1000 for BDI-II score; *t*(34) = 5.223, *p* < 0.001, Cohen's *d* = 1.741, BF_10_ > 1000 for DASS-A score. A significant positive correlation was noted between the BDI-II and DASS-A scores (*r* = 0.668, *p* < 0.001).

### Tasks

The study consisted of two tasks: a face-filtering change detection task and a VWM performance measurement task. The ability to filter face distractors from VWM was measured by EEG as the participants conducted the face-filtering task. VWM capacity was measured by another behavioral task (VWM performance measurement) because the VWM capacity of individuals can affect filtering ability (Owens et al., 2012; Vogel et al., 2005; Ye et al., 2018).

To ensure that the ERP results of the face-filtering task would not be influenced by the experience of the VWM performance measurement, all participants first completed the face-filtering task. The VWM performance measurement was then conducted on another day. Participants were seated in a dark room at a distance of 100 cm from a 17-inch screen when conducting these two tasks.

### Stimuli

**Figure 1.**
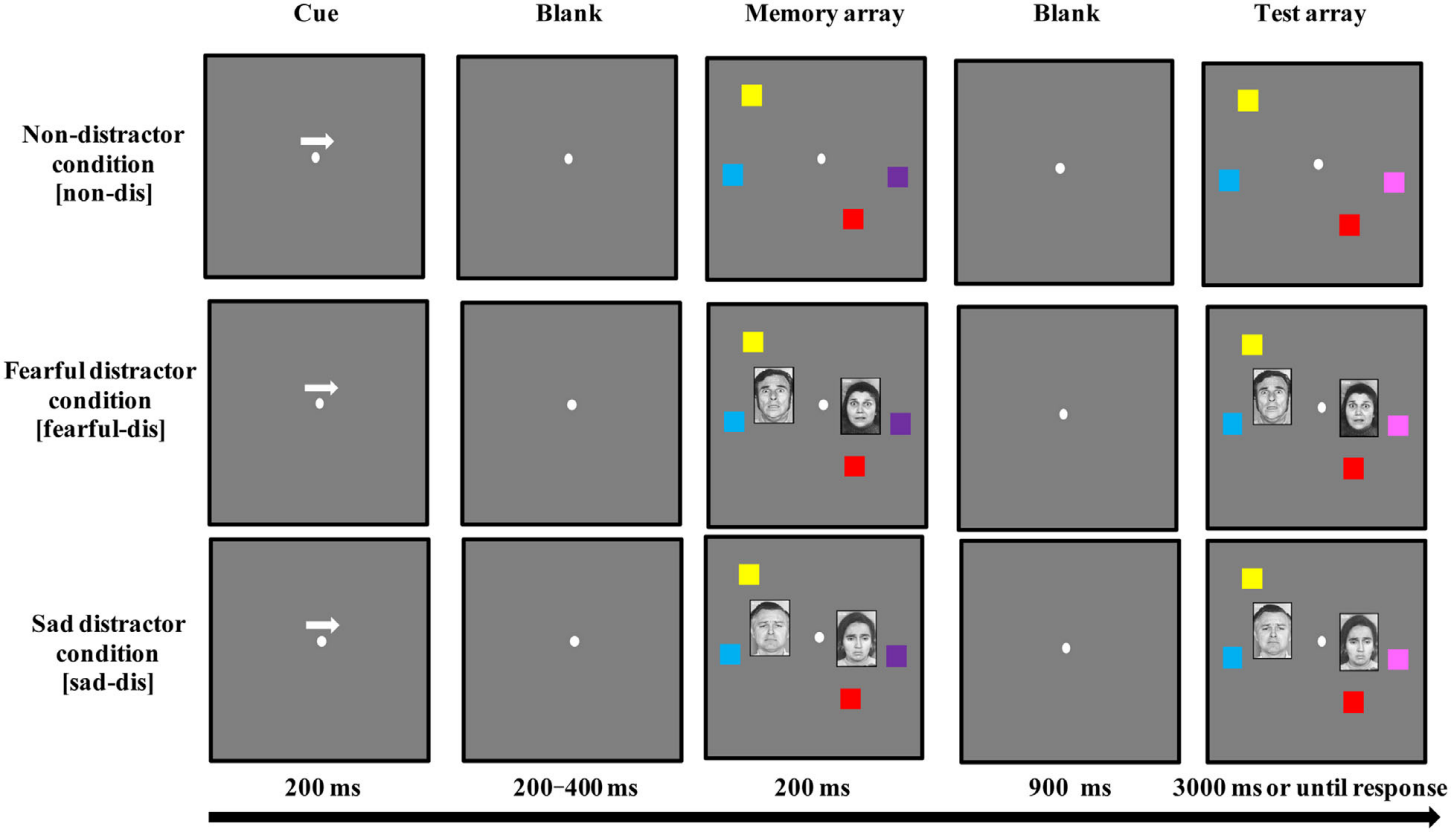
Trial structure showing the samples of three different conditions applied in the face-filtering task. Here, all arrow cues point to the right visual hemifield, and only trials with color changes are demonstrated.

For the face-filtering task, color squares (0.9° × 0.9°) and two different types of emotional (fearful and sad) face images (2.6° wide × 3° tall; black-and-white) were used as stimuli. The colors of the squares used as targets were selected randomly (without replacement) from a set of seven discriminable colors (red, green, blue, orange, yellow, purple, and pink). A total of 12 images (three fearful males, three fearful females, three sad males, and three sad females) used as distractors were selected from *Pictures of Facial Affect* (Ekman, 1976). The pictures were highly consistently classified as corresponding emotional faces (92.3% ± 0.05 for fearful images; 93.7% ± 0.04 for sad images). No significant difference was observed in classification accuracy between sad and fearful emotional images (*p* = 0.307). All colored squares and face images were presented bilaterally at random locations within 4° × 7.3° rectangular regions, centered 3° to the left and right of the center of the screen, against a gray background (see Figure 1). The positions of the stimuli were randomized in each trial and were separated by at least 2.6° (center to center).

For the VWM performance measurement, all stimulus arrays were presented against a gray background, and they occupied an area of 9.8° × 7.3°. Each item in the stimulus array was a square (0.65° × 0.65°) and had a randomly selected color without replacement from the set of seven discriminable colors (red, green, blue, orange, yellow, purple, and pink). The positions of the squares were randomized in each trial and separated by at least 2°.

### Experimental procedure

#### Face-filtering change-detection task

The face-filtering task was a lateralized color change-detection task with face distractors. The face-filtering task included three different conditions: non-distractor condition, fearful distractor condition, and sad distractor condition. As illustrated in Figure 1, each trial began with a fixation point (500 ms in duration) in the center of the screen, followed by a 200-ms arrow cue displayed above the fixation, pointing either to the left or right. After a variable interval (200–400 ms), a memory array, including four colored squares (two on the left hemifield and two on the right hemifield), was displayed for 200 ms. In the distractor conditions (fearful and sad distractor conditions), in addition to the colored squares, two emotional faces (one on the left hemifield and one on the right hemifield) were presented bilaterally in the memory array as distractors. Following the memory array, a blank screen (900 ms in duration) preceded the onset of the test array.

The test array was presented until the participants responded. The test array in the cued visual hemifield had one square of a different color compared to the memory array in 50% of the trials, whereas the memory array and test array were identical in all of the remaining trials. The change did not occur on either the face distractors or the color squares in the non-cued visual hemifield. Participants were informed that the colored squares displayed in the non-cued visual hemifield and all faces were irrelevant to the task. Participants were asked to memorize only the two-colored squares (as targets) in the cued hemifield, as indicated by the arrow cue. Each participant's task was to indicate whether the test array was identical to the memory array or whether one color had changed. The instruction emphasized response accuracy rather than response speed. Following the response, a variable interval (900–1100 ms) elapsed before the beginning of the next trial.

The non-distractor condition served as a baseline. In the non-distractor condition, two colored squares were presented on each side in the memory and test arrays, without any distractors. In the fearful distractor condition, a fearful face distractor was present, with two colored squares on each side, in both the memory and test arrays. Similarly, in the sad distractor condition, a sad face distractor was present, with two colored squares on each side, in both the memory and test arrays. The participants completed 200 trials for each condition (non-distractor, fearful-distractor, and sad-distractor), for a total of 600 trials, which were organized into 12 fully randomized blocks. A 30-second break occurred between each block. Twenty-four practice trials were given before the test performance was recorded. The entire task lasted approximately 60 minutes.

#### VWM performance measurement

**Figure 2.**
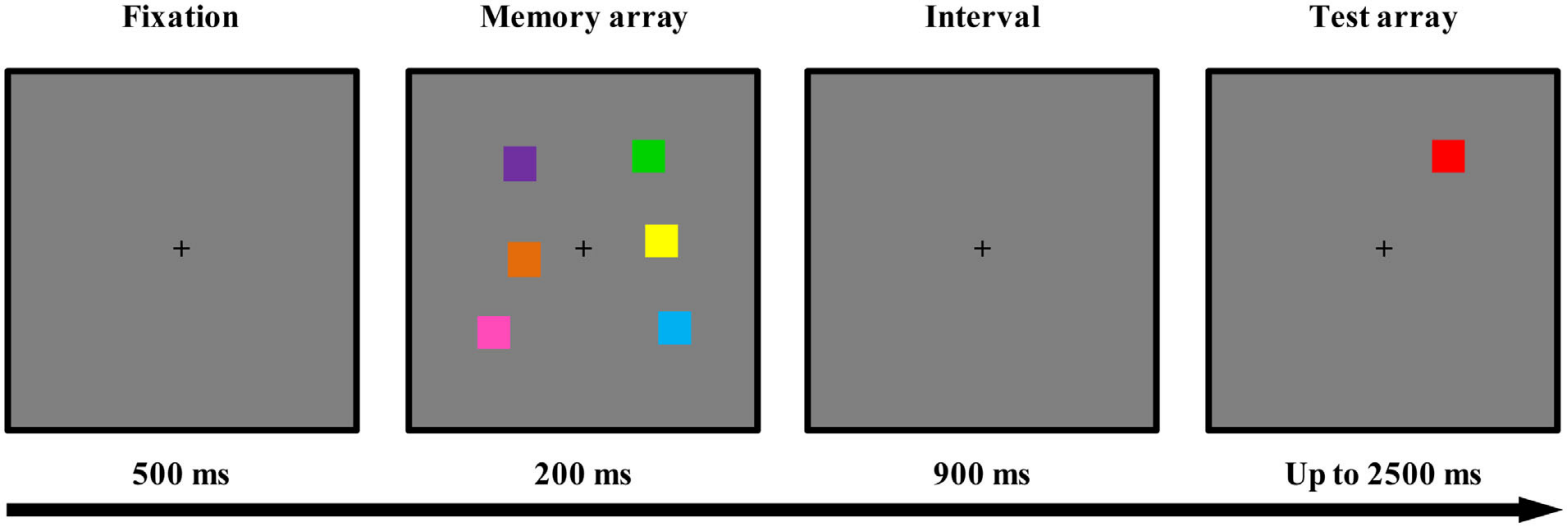
Trial structure of the VWM performance measurement. Here, only a trial with a change in the colored squares is demonstrated, but trials that had no changes were also run.

As illustrated in Figure 2, each trial began with a 500-ms fixation cross, followed by a sample array of six colored squares (presented for 200 ms). After a blank interval (900 ms), a probe array with one colored square (2500 ms) was presented. The participants needed to indicate whether the probe color was the same as the one in that specific location in the memory array, with accuracy rather than response speed being stressed. The probe color was different from that in the memory array in 50% of the trials and was identical in the remaining trials. All participants completed 100 trials of this task, with a 30-second break after the first 50 trials. The measurement lasted approximately 10 minutes. No EEG measurements were made during the VWM performance measurements.

### EEG recording and analyses

Continuous EEG was measured and amplified with a NeurOne system (Bittium Biosignals Ltd., Kuopio, Finland), and a 128-channel net (HydroCel Geodesic Sensor Net, Electric Geodesic Inc., Eugene, OR) was applied. EEG signals were recorded by online referencing to the vertex electrode (Cz) in AC mode. The data were bandpass filtered (0.1–250 Hz) and sampled at 1000 Hz. Upon arrival at the EEG laboratory, each participant was fitted with an EEG cap of the appropriate size. Four EOG electrodes were placed vertically (above and below the right eye) and horizontally (next to each eye) to measure eye movements during the task. All participants were instructed to sit as still and as relaxed as possible and not blink excessively.

The data were analyzed offline with a BrainVision Analyzer 2.1 (Brain Products GmbH, Munich, Germany). Topographic interpolation was first applied to estimate the amplitude of bad electrodes from neighboring electrodes. An average was calculated over all channels to serve as a new reference. Based on our previous studies (Ye, Zhang, Liu, Li, & Liu, 2014; Ye et al., 2018), the averaged ERP waveforms were filtered by applying a 17-Hz low-pass filter. The EEG was segmented into 1300-ms epochs, starting from 200 ms before the onset of the memory array. The epochs were baseline corrected for the 200-ms pre-stimulus interval.

Based on previous studies (Sessa et al., 2011; Stout et al., 2013), the trials contaminated with extensive horizontal eye movements (which were reflected by horizontal electrooculogram [HEOG] amplitudes greater than ±60 µV) were excluded from the analysis. Any trials with remaining artifacts exceeding ±80 µV in amplitude were also rejected. Participants with trial rejection rates higher than 30% were excluded from the analyses.

We also explored the differences in the number of rejected trials caused by excessive horizontal eye movements between different groups and conditions by conducting a repeated-measures analysis of variance (ANOVA) with the condition (non-distractor vs. fearful distractor vs. sad distractor), attending hemifield (left-attending vs. right-attending), and participant group (depressed vs. control) for the trial rejection rates due to the exclusion criterion of the HEOG. No significant main effect was found for condition, encoding hemifield, or participant group (all *p* > 0.263), and no significant interaction was evident between condition, encoding hemifield, and participant group (all *p* > 0.126). These results suggest that the participant group and condition had no significant impact on the number of excluded trials due to extensive eye movements.

Based on previous studies (McCollough, Machizawa, & Vogel, 2007; Ye et al., 2018), we chose three pairs of electrodes at the posterior parietal sites (P7/8, P9/10, and PO7/8) for analysis. For each condition, the contralateral waveforms were calculated by averaging the activity recorded at the left hemisphere electrode sites when the participants were cued to memorize the right side of the memory array, and with the activity recorded at right hemisphere electrode sites when they were cued to memorize the left side. The ipsilateral waveforms were computed by averaging the left and right hemisphere sites when the participants were cued to memorize the left and right sides of the memory array, respectively. The CDA amplitude was defined by subtracting the ipsilateral activity from the contralateral activity at a measurement window of 500 to 1000 ms after the onset of the memory array.

Considering that there are different processing mechanisms in the early and late phases of VWM consolidation (Long, Ye, Li, Tian, & Liu, 2020; Ye, Hu, Li, Ristaniemi, Liu, & Liu, 2017; Ye, Liang, Zhang, Xu, Zhu, & Liu, 2020; Ye, Sun, Xu, Liang, Zhang, & Liu, 2019), in addition to the CDA component, we conducted exploratory analyses for other early contralateral activities (e.g., the positivity posterior contralateral [Ppc] and N2pc components). More details of their results and discussion can be found in the Supplementary Materials.

### VWM performance measurement analysis

Previous studies have often used *K* values to measure VWM capacity (Vogel et al., 2005; Ye et al., 2018), but other indexes (e.g., *d*′) have recently been suggested as potentially more valid measures of VWM in change detection tasks (Williams, Robinson, Schurgin, Wixted, & Brady, 2022). Therefore, for the VWM performance measurement, we analyzed both the *K* value and *d*′ (i.e., sensitivity), which can reflect the VWM performance of the participants. We performed independent sample *t*-tests to compare the *K* value and *d*′ of participants in the control group to those in the depression group. We have shared these behavioral results (including the actual hit rates, false alarm rates, *K* value, and *d*′) on the Open Science Framework (https://osf.io/casz7/).

#### *K* value

The VWM capacity of each participant was quantified based on the results of their VWM performance measurements. We used the standard formula proposed by Cowan (2001): *K* = *N* × (*H* − *F*), where *K* is the VWM capacity, *N* is the size of the array (i.e., six in the present study), *H* is the hit rate or proportion of correct responses when a change is present, and *F* is the false alarm rate or proportion of incorrect responses when no change is present.

#### Calculation of *d*′

The *d*′ score, which represents the sensitivity in the VWM performance measurement, was calculated as the difference between hit rates and false alarm rates: *d*′ = *Z*(hit rate) – *Z*(false alarm).

### Statistical analysis

For the face-filtering change-detection task, we conducted separate repeated-measures ANOVA, with condition (non-distractor vs. fearful distractor vs. sad distractor) as a within-subject factor and participant group (depressed vs. control) as a between-subject factor for the behavioral performance (i.e., accuracy) and amplitude of the ERP components. Partial eta squared (η*_p_*^2^) measures were used for the effect size estimations of the ANOVAs. For the planned comparison tests, we conducted paired *t*-tests to compare the results between different conditions in both groups, and an independent samples *t*-test to compare the results between groups under different conditions. The VWM capacity between the groups was compared using an independent samples *t*-test. Cohen's *d* was used as an estimator of the effect size of significant results in the t-tests. We used JASP 0.16 to conduct Bayes factor analyses (Bayesian *t*-test) to show whether the *t*-test results supported the alternative hypothesis or the null hypothesis (Rouder, Speckman, Sun, Morey, & Iverson, 2009). The default priors in JASP were used (Schmalz, Biurrun Manresa, & Zhang, 2021). The Bayes factor (BF_10_) provides an odds ratio for alternative/null hypotheses (values < 1 favor a null hypothesis and values > 1 favor an alternative hypothesis); for example, a BF_10_ of 0.25 would indicate that acceptance of the null hypothesis is four times more likely than acceptance of the alternative hypothesis. The datasets generated and analyzed during this study and experimental scripts are available online via the Open Science Framework (https://osf.io/casz7/).

## Results

### VWM performance measurement (*K* value and *d*′)

The *K* value results showed no significant difference between the control (*K* = 2.66, *SD* = 1.005) and depressed groups (*K* = 2.42, *SD* = 0.688), *t*(34) = 0.836, *p* = 0.409, Cohen's *d* = 0.279, BF_10_ = 0.423. The VWM capacity showed no significant correlation with the BDI-II scores (*r* = –0.134, *p* = 0.438) or with the DASS-A scores (*r* = 0.007, *p* = 0.967).

The *d*′ results showed no significant difference between the control group (*d*′ = 1.61, *SD* = 0.433) and depressed group (*d*′ = 1.40, *SD* = 0.590), *t*(34) = 1.244, *p* = 0.318, Cohen's *d* = 0.406, BF_10_ = 0.586. The VWM capacity showed no significant correlation with the BDI-II scores (*r* = –0.258, *p* = 0.128) or with the DASS-A scores (*r* = –0.102, *p* = 0.553). The result pattern obtained for the *d*′ was consistent with that of the *K* value.

### Accuracy

The ANOVA for the accuracy of the responses showed a significant main effect of condition, *F*(2, 68) = 12.578, *p* < 0.001, η*_p_*^2^ = 0.270, but no significant main effect of group, *F*(1, 34) = 0.100, *p* = 0.754, η*_p_*^2^ = 0.003) or significant interaction of condition by group, *F*(2, 68) = 0.963, *p* = 0.387, η*_p_*^2^ = 0.028.

**Figure 3.**
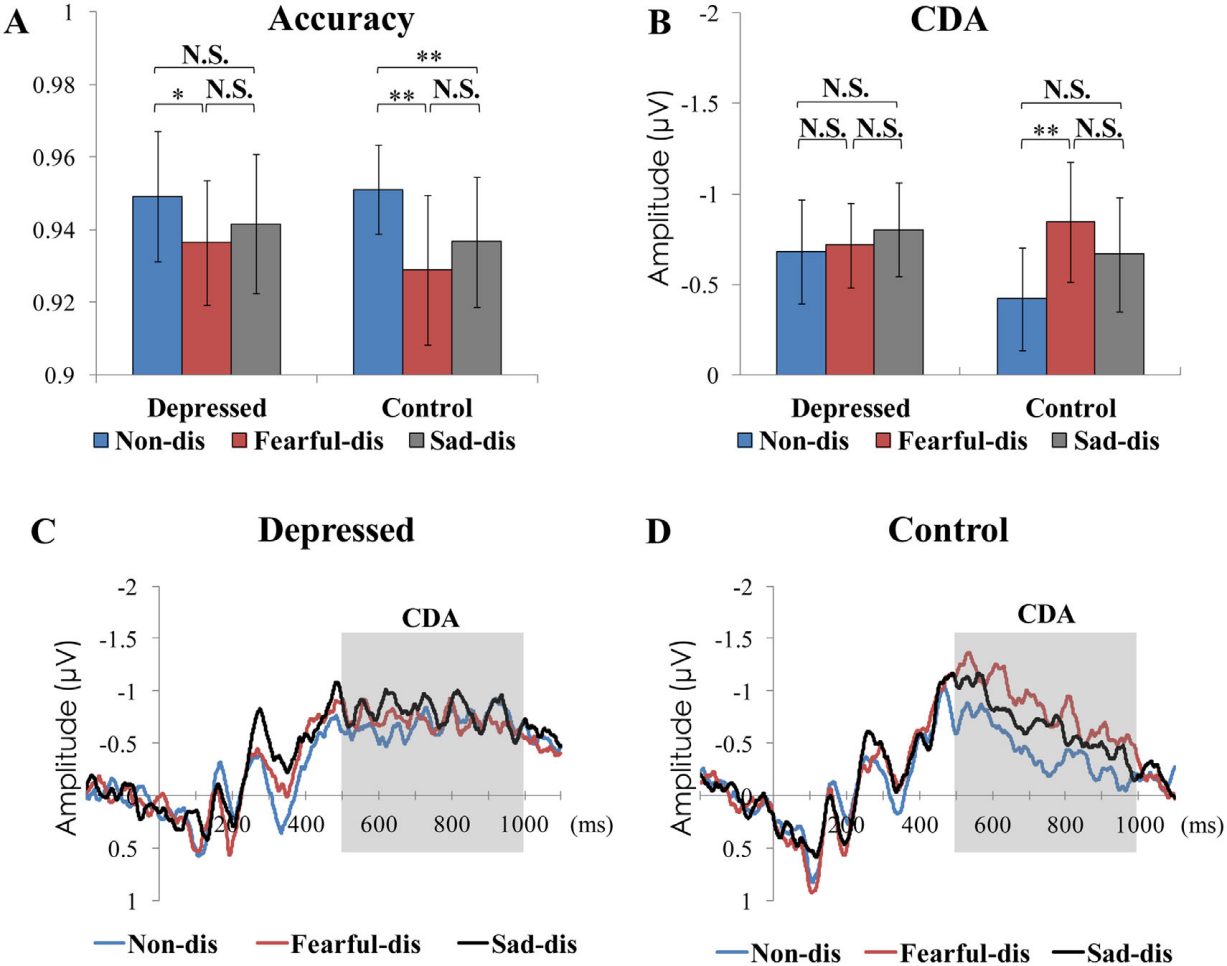
Behavioral and CDA results. (A) The accuracy results (mean and standard error of mean) for depressed (left) and control (right) groups separately under different conditions. (B) The results of the CDA amplitude for the depressed (left) and control (right) groups under different conditions are shown separately. Bars show the mean values, and their error bars depict the 95% confidence interval of the mean. **p < 0.01; *p < 0.05; N.S., non-significant (*p* > 0.05). (C) Difference waves (contralateral waves minus ipsilateral waves) of grand average ERPs (averaged over P7/P8, P9/P10, and PO7/PO8) under different conditions elicited by memory arrays for the depressed group. Gray shades indicate the analysis time window used to calculate the mean CDA amplitude. The waveforms are time locked to the onset of the memory array (*y*-axis on time zero). (D) Difference waves of the grand average ERPs (averaged over P7/P8, P9/P10, and PO7/PO8) under different conditions elicited by memory arrays for the control group. Non-dis = non-distractor condition, Fearful-dis = fearful distractor condition, Sad-dis = sad distractor condition.

The accuracy values and results are presented in Table 1 and Figure 3A. The planned comparisons showed a higher accuracy for the depressed group in the non-distractor condition than in the fearful distractor condition, *t*(17) = 2.848, *p* = 0.011, Cohen's *d* = 0.363, BF_10_ = 4.838, but no significant difference in accuracy was found between the non-distractor condition and the sad distractor condition, *t*(17) = 1.751, *p* = 0.098, Cohen's *d* = 0.201, BF_10_ = 0.865, or between the fearful distractor condition and the sad distractor condition, *t*(17) = 1.329, *p* = 0.201, Cohen's *d* = 0.145, BF_10_ = 0.518. For the control group, the accuracy was higher in the non-distractor condition than in the fearful distractor condition, *t*(17) = 3.369, *p* = 0.004, Cohen's *d* = 0.649, BF_10_ = 12.341, or in the sad distractor condition, *t*(17) = 3.010, *p* = 0.008, Cohen's *d* = 0.465, BF_10_ = 6.447, but no significant difference in accuracy was found between the fearful distractor condition and sad distractor condition, *t*(17) = 1.458, *p* = 0.163, Cohen's *d* = 0.201, BF_10_ = 0.598.

**Table 1.** Mean values and standard deviations (in parentheses) for behavioral accuracies, CDA amplitudes, and CDA difference scores under each condition for the depressed and control groups. *Notes*: Non-dis = non-distractor condition; Fearful-dis = fearful distractor condition; Sad-dis = sad distractor condition.

	Depressed			Control		
Condition	Accuracy	CDA amplitude (µV)	CDA difference score (µV)	Accuracy	CDA amplitude (µV)	CDA difference score (µV)
Non-dis	94.92% (0.04)	−0.68 (0.58)	—	95.11% (0.02)	−0.42 (0.57)	—
Fearful-dis	93.64% (0.03)	−0.71 (0.47)	−0.03 (0.36)	92.89% (0.04)	−0.85 (0.67)	−0.43 (0.55)
Sad-dis	94.17% (0.04)	−0.80 (0.52)	−0.12 (0.36)	93.67% (0.04)	−0.67 (0.57)	−0.25 (0.60)

We also compared the accuracy between the depressed group and the control group under each condition. No significant group difference was noted under the non-distractor condition, *t*(34) = 0.188, *p* = 0.852, Cohen's *d* = 0.061, BF_10_ = 0.326; under the fearful distractor condition, *t*(34) = 0.590, *p* = 0.559, Cohen's *d* = 0.197, BF_10_ = 0.369; or under the sad distractor condition, *t*(34) = 0.402, *p* = 0.690, Cohen's *d* = 0.134, BF_10_ = 0.343.

### Contralateral delay activity

The grand-averaged difference waveforms (contralateral waveforms minus ipsilateral waveforms; the contralateral and ipsilateral waveforms are provided in Supplementary Figure S1), and the histograms showing their CDA amplitude values are depicted separately for the depressed and control groups in Table 1 and Figures 3B to 3D. For the CDA amplitude, the ANOVA showed a significant main effect of condition, *F*(2, 68) = 5.204, *p* = 0.008, η*_p_*^2^ = 0.133, and a significant interaction of condition by group, *F*(2,68) = 3.530, *p* = 0.035, η*_p_*^2^ = 0.094, but no significant main effect of group, *F*(1, 34) = 0.271, *p* = 0.606, η*_p_*^2^ = 0.008.

The planned comparisons investigating the condition × group interaction are reported in Table 2. The depressed group showed no significant differences in CDA amplitude between the different conditions. By contrast, the control group showed a higher CDA amplitude in the fearful distractor condition than in the non-distractor condition. No significant difference was detected for the CDA amplitude between the non-distractor and sad distractor conditions or between the sad distractor and fearful distractor conditions.

**Table 2.** Results of the follow-up paired-samples *t*-tests investigating the interaction of condition × group for CDA amplitudes separately in the depressed group and in the control group. *Notes*: Non-dis = non-distractor condition; Fearful-dis = fearful distractor condition; Sad-dis = sad distractor condition; df = degrees of freedom; *d* = Cohen's *d*; **p < 0.01.

	Depressed					Control				
Conditions	df	*t*	*p*	*d*	BF_10_	df	*t*	*p*	*d*	BF_10_
Non-dis vs. Fearful-dis	17	0.404	0.691	0.006	0.262	17	3.293	0.004^**^	0.692	10.724
Non-dis vs. Sad-dis	17	1.452	0.165	0.222	0.594	17	1.761	0.096	0.410	0.876
Fearful-dis vs. Sad-dis	17	0.933	0.364	0.177	0.356	17	1.885	0.077	0.277	1.038

We also compared the amplitudes of the CDA between the depressed group and the control group under each condition. No significant group difference was noted in the CDA amplitude under the non-distractor condition, *t*(34) = 1.376, *p* = 0.178, Cohen's *d* = 0.459, BF_10_ = 0.669; the fearful distractor condition, *t*(34) = 0.684, *p* = 0.499, Cohen's *d* = 0.228, BF_10_ = 0.386; or the sad distractor condition, *t*(34) = 0.707, *p* = 0.485, Cohen's *d* = 0.236, BF_10_ = 0.391.

Previous studies have suggested that VWM capacity can affect the unnecessary memory storage of distractors (Owens et al., 2012; Vogel et al., 2005; Ye et al., 2018); therefore, we used the VWM capacity as a covariant in a repeated measures analysis of covariance (ANCOVA) for the CDA amplitude (as in the original analysis, where condition was a within-subject variable and participant group was a between-subject variable). This analysis, which controls for VWM capacity, showed results similar to those for the original significant interaction of condition by group, *F*(2, 66) = 3.385, *p* = 0.040, η*_p_*^2^ = 0.093.

We also examined whether depressive symptoms (BDI-II scores) or anxiety symptoms (DASS-A scores) affected VWM resource allocation to distractors. We first calculated the mean CDA amplitude difference scores between the distractor condition and the non-distractor (baseline) condition for both the fearful and sad face distractors (i.e., CDA amplitude in fearful/sad distractor condition minus CDA amplitude in the non-distractor condition). The occurrence of a CDA difference score with a negative value indicates a larger CDA in the distractor condition compared to the non-distractor condition, suggesting that the participants have difficulty filtering the distractors. The results of the CDA difference scores for the depressed and control groups are presented in Table 1. The CDA difference scores for fearful face distractors were significantly larger in the control group than in the depressed group, *t*(34) = 2.547, *p* = 0.016, Cohen's *d* = 0.849, BF_10_ = 3.589, but no significant difference was found for the CDA difference scores for sad face distractors between the control group and the depressed group, *t*(34) = 0.763, *p* = 0.451, Cohen's *d* = 0.254, BF_10_ = 0.404. Moreover, the correlation results over the whole sample for the VWM capacity showed no significant correlation between the *K* value and the CDA difference scores for fearful face distractors (*r* = –0.154, *p* = 0.371) or sad face distractors (*r* = –0.282, *p* = 0.096). The results of the depressive symptoms showed a significant positive correlation between the BDI-II scores and the CDA difference scores for the fearful face distractors (*r* = 0.357, *p* = 0.032). No significant correlation was found between the BDI-II scores and the CDA difference scores for the sad face distractors (*r* = 0.158, *p* = 0.357). The results for the anxiety symptoms showed no significant correlation between the DASS-A scores and the CDA difference scores for the fearful face distractors (*r* = 0.262, *p* = 0.123) or for the sad face distractors (*r* = 0.140, *p* = 0.417).

## Discussion

This study investigated whether non-depressed and depressed participants could filter fearful and sad face distractors during a color-change detection task. Our main result, indicated by the CDA amplitude, was that the control group failed to filter fearful face distractors from VWM, whereas the depressed group showed no difficulty in this filtering. Sad face distractors did not consume additional VWM resources in either the control or the depressed groups.

The CDA results in the control group were well in line with previous findings regarding the unnecessary storage of fearful face distractors in VWM (Stout et al., 2013). In accordance with our expectations, the CDA results suggest that the control participants were able to filter sad face distractors from VWM. This is a novel result, as previous CDA studies have not applied sad face distractors (Stout et al., 2013; Ye et al., 2018; Zhang et al., 2021). Our findings suggest that non-depressed individuals do not store all negative facial distractors in VWM; rather, they selectively and automatically store potentially dangerous signals (i.e., fearful face distractors), even if the distractors are task irrelevant. This finding is congruent with many studies demonstrating that the processing of threat-related stimuli is prioritized in many ways in the human brain (LeDoux, 1996). Threatening faces are detected more rapidly (Eimer & Holmes, 2002; Ohman, Lundqvist, & Esteves, 2001; Schupp, Ohman, Junghofer, Weike, Stockburger, & Hamm, 2004; Xu et al., 2021), and the change-detection brain responses they elicit occur earlier than those elicited by other facial expressions (Astikainen & Hietanen, 2009; Bayle & Taylor, 2010; Smith, Cacioppo, Larsen, & Chartrand, 2003; Stefanics, Csukly, Komlosi, Czobor, & Czigler, 2012). Threatening stimuli are also more arousing than non-threatening stimuli (Posner, Russell, & Peterson, 2005). Therefore, the control group's difficulty filtering fearful faces could be explained by the high arousal triggered by the fearful face distractors. Future studies should be conducted to investigate this possibility by manipulating the intensity (and thus arousal) of facial emotions in distractors.

We expected to observe a similar pattern of results in both the control and depressed groups regarding the ability to filter fearful faces. However, we found no difference in the CDA amplitude between the conditions in the depressed group, suggesting that the fearful face distractors did not consume additional VWM resources in depressed participants. The group difference in fearful face filtering, as well as the correlation results, indicated a reduction in the VWM resources occupied by fearful face distractors among participants with depressive symptoms. One possibility is that the increased efficacy in filtering fearful face distractors in the depressed group is due to their decreased overall responsiveness to emotional stimuli (i.e., emotion context insensitivity) (Bylsma, Morris, & Rottenberg, 2008; Rottenberg, Gross, & Gotlib, 2005; Rottenberg & Hindash, 2015), and this could result in less interest in threatening human faces in the depressed group than in the control group.

One noteworthy finding was that the behavioral accuracy of depressed participants was significantly lower under the fearful distractor condition than under the non-distractor condition. This result suggests that the appearance of fearful face distractors indeed impaired the task performance of depressed participants. However, because the CDA was not affected, the pattern of the results suggests that the impaired behavioral performance may be due to alterations in the decision-making phase after the appearance of the test array rather than in the VWM maintenance phase, which the CDA reflects. In other words, the face distractors in the test array may attract the attention of the participants and impair the processing of probe colors. However, because the CDA is extracted from the time window before the test array appears, our ERP results may not reflect alterations in the behavioral level. Therefore, the results of the present study should not be interpreted simply as depressive symptoms enhancing an individual's ability to filter fearful face distractors.

Our recent study indicated that participants with a high VWM capacity could filter both neutral face distractors and negative face distractors from VWM, whereas those with a low VWM capacity failed to filter either of them (Ye et al., 2018). However, similar to another study that have not found the relationship between VWM capacity and internal attention ability(Ye et al., 2021), in the present study, we found no significant correlation between VWM capacity (i.e., *K* value) and filtering efficiency (i.e., CDA difference scores); therefore, the present results seem to be inconsistent with those of our previous study (Ye et al., 2018). The most plausible reason for this inconsistency could be that our experimental design differed from that used in previous studies. For example, in our previous study (Ye et al., 2018), our memory targets and distractors were the same kinds of stimuli (i.e., faces). Thus, the participants needed to spend additional resources selecting the targets and filtering the distractors. However, in the present study, the distractors (i.e., faces) were completely different from the memory targets (i.e., squares in different colors), which enabled the participants to easily identify and select memory targets without spending additional resources. Therefore, in the present design, filtering the distractors was easier, and identifying the targets took less effort because the distractors were not similar to the targets.

These findings raise the possibility that the correlation between the VWM capacity and the filtering efficiency is observable only when the memory targets are similar to the distractors and are therefore difficult to identify. The present result is consistent with that of another previous study on the relationship between VWM capacity and the distractor capture attention effect (Fukuda & Vogel, 2009). Fukuda and Vogel (2009) found a strong positive correlation between the VWM capacity and the resistance to attentional capture from distractors when the distractors were similar to the targets, but this positive correlation was not observed for trials with dissimilar distractors. Their results indicated that the relationship between VWM capacity and the distractor capture attention effect is restricted to capture attention triggered by the presence of distractors that are highly similar to the target. Thus, when memory targets are not similar to distractors, the correlation between VWM capacity and filtering efficiency disappears. Other recent studies have also suggested that filtering efficiency is modulated by the target–distractor similarity (Liesefeld, Liesefeld, Sauseng, Jacob, & Müller, 2020; Williams & Drew, 2021).

We also found that the depressed participants in the present study did not store distractors (e.g., fearful faces) in VWM. These results may also be due to the setup of the dissimilarity between memory targets and distractors. One possibility is that when the memory targets and distractors are the same kinds of stimuli, we can also observe that depressed participants have filtering efficiency deficits for certain emotional distractors. Future research should consider the effects of the same/similar kind of memory targets and distractors to examine the distractor filtering process during the VWM maintenance of depressed participants.

Interestingly, the CDA results were not completely the same as the behavioral results. Notably, the CDA and behavioral results are indexes of different aspects of the task: Whereas CDA is an index of the number of items in the VWM during the maintenance phase, the behavioral responses indicate only the end result of the task without separating the different phases (e.g., memory encoding, maintenance of visual information, memory retrieval, decision making). When we analyzed the ERP data, we set the analysis time window between the onset of the memory array and the onset of the test array. This setup ensured that our ERP results were not affected by the test array content of each trial. By contrast, the behavioral results could be affected by the content and decision-making phase occurring in the test array. This meant that the behavioral results included the impact of other variables, which caused inconsistencies between the CDA and behavioral results.

The present study has some potential limitations. Recent studies have shown that anxiety symptoms impair VWM capacity, VWM processing efficiency, and distractor filtering ability (Song et al., 2021; Song, Chang, & Zhou, 2022; Stout & Rokke, 2010). Some research has demonstrated that anxious participants show particularly inefficient filtering of fearful face distractors from VWM (Stout et al., 2013; Stout et al., 2015; Stout et al., 2017). In our study, four participants in the depressed group had a comorbid anxiety disorder, and anxiety symptoms were significantly higher in the depressed group than in the control group. Previous studies suggest that depression and anxiety are highly comorbid (Hirschfeld, 2001). We also found a significant positive correlation between anxiety symptoms and depression symptoms; therefore, finding participants who had only depression and no anxiety symptoms was difficult. Nonetheless, we did not find any correlation between the participants’ anxiety symptoms (DASS-A) and filtering efficiency in the fearful distractor condition (CDA difference scores for fearful distractors). In addition, anxiety symptoms would not explain the group differences detected for fearful face filtering in the present study, because anxiety should increase (Stout et al., 2013) rather than decrease the difficulty in filtering fearful face distractors. Therefore, this limitation should not weaken the value of the findings of this study. However, our sample size is clearly small to allow for correlation analyses (Button et al., 2013; Dubois & Adolphs, 2016). Therefore, the results of our correlation analyses can only be considered exploratory, and any correlation should be interpreted with caution.

Another potential limitation of our experimental design is that the visual array size of the memory array in the non-distractor condition (four colors) was not equal to that in the distractor conditions (four colors and two faces). The stimulus-driven factor (e.g., visual array size) may also potentially affect resource allocation and distractor filtering mechanisms. For example, a larger visual array size may require more cognitive resources for visual encoding. Thus, in future research, a neutral distractor condition could be applied as the baseline (e.g., four color targets and two neutral face distractors) to compare with the emotional distractor conditions. In our study, however, the main focus was on a comparison of the two negative face distractors (sad and fearful) and the two groups (depressed and controls).

Previous research has demonstrated that VWM performance is worse when visual items are allocated within only one hemifield than in both the left and right visual fields (Delvenne, 2005; Umemoto, Drew, Ester, & Awh, 2010), and this is attributed to the allocation of fewer attentional resources (Zhang et al., 2018). However, as in many previous studies (Owens et al., 2012; Stout et al., 2013; Vogel et al., 2005; Ye et al., 2018), and in the present study, the traditional VWM experimental designs for distractor filtering present the distractors and targets in the same visual hemifield. This setup complicates any disentanglement of the contribution of the target and distractor to the elicited ERP activity. Although we observed an increased CDA amplitude in the distractor conditions (e.g., the fearful face distractor condition), our design cannot identify whether the mechanism underlying the enhanced CDA amplitude in distractor conditions involves an increase in memory storage, a decrease in memory suppression, or some combination of these. The Ppc and N2pc findings reported in the Supplementary Materials have the same issue (they cannot resolve the issue with distractor suppression vs. attentional enhancement) because of this experimental design. Further, the cause of VWM storage exists even prior to the time window of CDA (e.g., during an early stage of attentional selection). Therefore, the CDA results in our study should not be considered a direct measure of distractor filtering ability; rather, they are a measure of the stored information after the participant has undergone attentional selection and distractor filter processes.

One reasonable assumption is that enhanced VWM storage for the distractors (e.g., fearful faces) reflects enhanced attention to the distractors (e.g., fearful faces), as shown by increased N2pc (Salahub & Emrich, 2020) and amygdala activation (Stout et al., 2017). In the present study, our results also showed a positive correlation between the N2pc difference scores and the CDA difference scores (for more detailed results, see Supplementary Materials), which is in line with the findings reported by Salahub and Emrich (2020). This raises the question of which processes determine whether distractors will be stored in VWM. Further investigation of this question will require isolating the attention and memory processes for distractors from those for memory targets.

Recent studies have used a novel paradigm to investigate this particular question with simple neutral items (Feldmann-Wustefeld & Vogel, 2019) or neutral/fearful faces (Salahub & Emrich, 2020). The contribution of active suppression is investigated by presenting the targets or the distractors on the lateral field or on the vertical midline (targets on the lateral/distractors on the vertical midline, or distractors on the lateral/targets on the vertical midline). This setting allows researchers to isolate the attention and suppression processes of targets or distractors. Future studies could use a similar paradigm to investigate the mechanism of other emotional faces (e.g., angry or happy) in distractor filtering.

In summary, our results indicate that non-depressed individuals have difficulty filtering fearful task-irrelevant information from VWM, even if the target selection is simplified by the use of different types of targets and distractors. By contrast, in depressed individuals, fearful task-irrelevant information does not consume VWM resources. Further, sad face distractors did not consume additional VWM resources either in the non-depressed individuals or, unexpectedly, in the depressed individuals. Additional studies are needed to obtain a better understanding of the cognitive and neural mechanisms underlying the effect of depressive symptoms on the ability to filter task-irrelevant emotional information.

## Supplementary Material

Supplement 1
