# Supplementary material for: Alterations in working memory maintenance of fearful face distractors in depressed participants: An ERP study
Source: J Vis. 2023 Jan 18;23(1):10. doi: 10.1167/jov.23.1.10 (PMC9855285; doi:10.1167/jov.23.1.10)
Supplement: Supplement 1 [file jovi-23-1-10_s001.pdf]

Supplementary Materials

Alterations in working memory maintenance of fearful face distractors in depressed participants: An ERP study

Chaoxiong Ye[1,2,3,4], Qianru Xu[3], Xueqiao Li[2], Elisa Vuoriainen[4], Qiang Liu[1*], Piia Astikainen[2*]

[1] Institute of Brain and Psychological Sciences, Sichuan Normal University, Chengdu, China;
[2] Department of Psychology, University of Jyvaskyla, Jyväskylä, Finland;
[3] Center for Machine Vision and Signal Analysis, University of Oulu, Oulu, Finland;
[4] Faculty of Social Sciences, Tampere University, Tampere, Finland.

*Correspondence should be addressed to Qiang Liu, Institute of Brain and Psychological Sciences, Sichuan Normal University, Chengdu, 610068, China. E-mail: lq780614@163.com; and Piia Astikainen, Department of Psychology, University of Jyvaskyla, P.O. Box 35, 40014 Jyväskylä, Finland. E-mail: piia.astikainen@jyu.fi.

1. Contralateral and ipsilateral waveforms

The difference waveforms for the CDA component in the main text were determined by calculating the mean contralateral and ipsilateral ERP activity recorded at the electrode pool P9/10, P7/P8, and PO7/8. Here, we provide the separate contralateral and ipsilateral waveforms for each condition, for each participant group, and separately for sites contra- and ipsilateral to the cued hemifield, resulting in the six pairs of waveforms shown in Fig. S1.

The difference waveforms for the Ppc and N2pc components in the main text were determined by calculating the mean contralateral and ipsilateral ERP activity recorded at electrode pool PO7/8. Here, we provide the separate contralateral and ipsilateral waveforms for each condition and separately for sites contra- and ipsilateral to the cued hemifield, resulting in the three pairs of waveforms shown in Fig. S2.

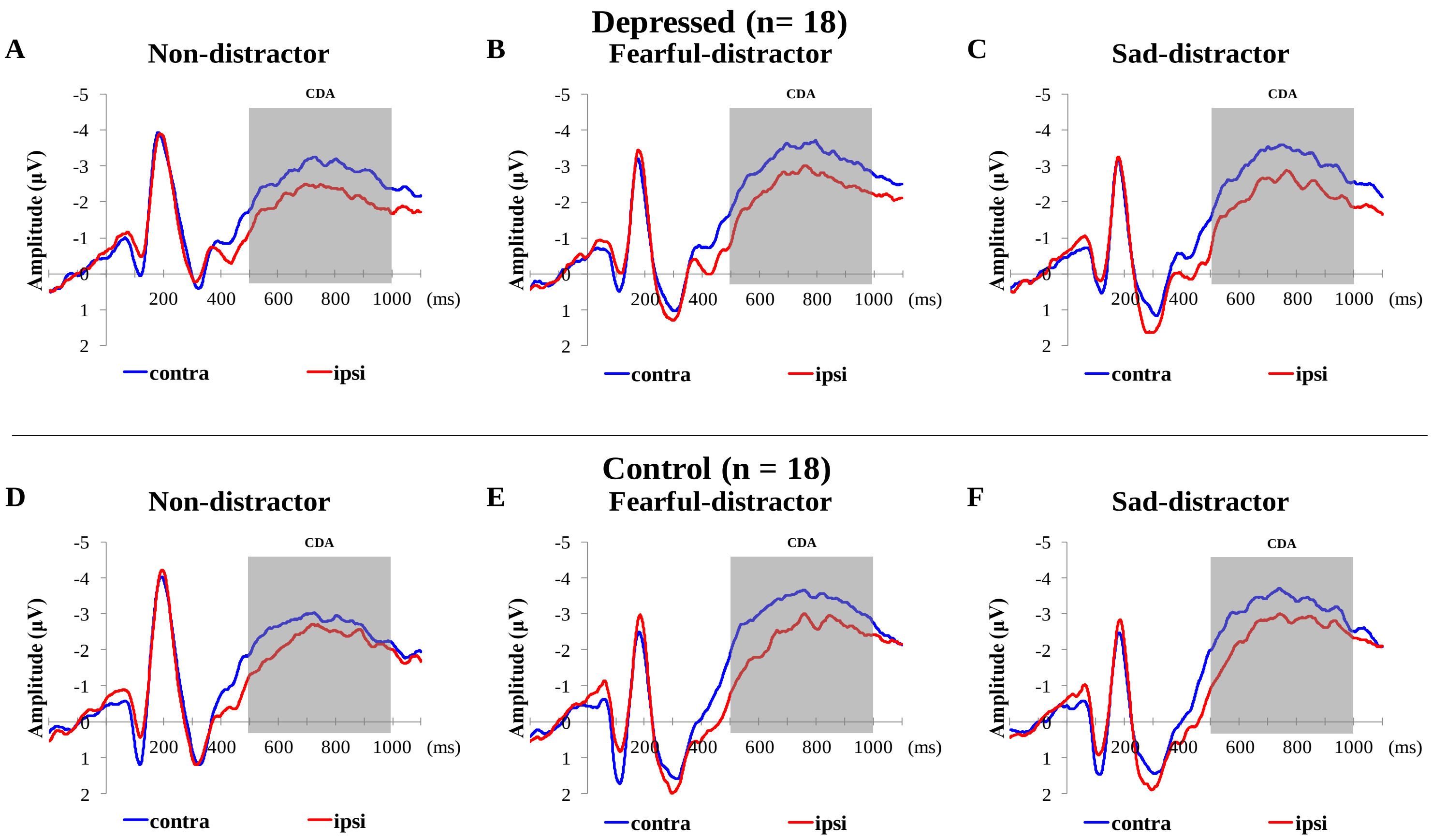

Fig. S1 Grand average ERPs (averaged over P7/P8, P9/P10, and PO7/PO8) elicited by memory arrays under (A, D) the non-distractor condition, (B, E) the fearful distractor condition, and (C, F), the sad distractor condition for the (A–C) depressed group and (D–F) control group. Blue lines reflect the activity contralateral to the lateralized items, and red lines reflect the activity ipsilateral to the lateralized items. Gray shades indicate the analysis time window used to calculate the mean CDA amplitude. The waveforms were time-locked to the onset of the memory array (y-axis on time zero). contra = contralateral, ipsi = ipsilateral

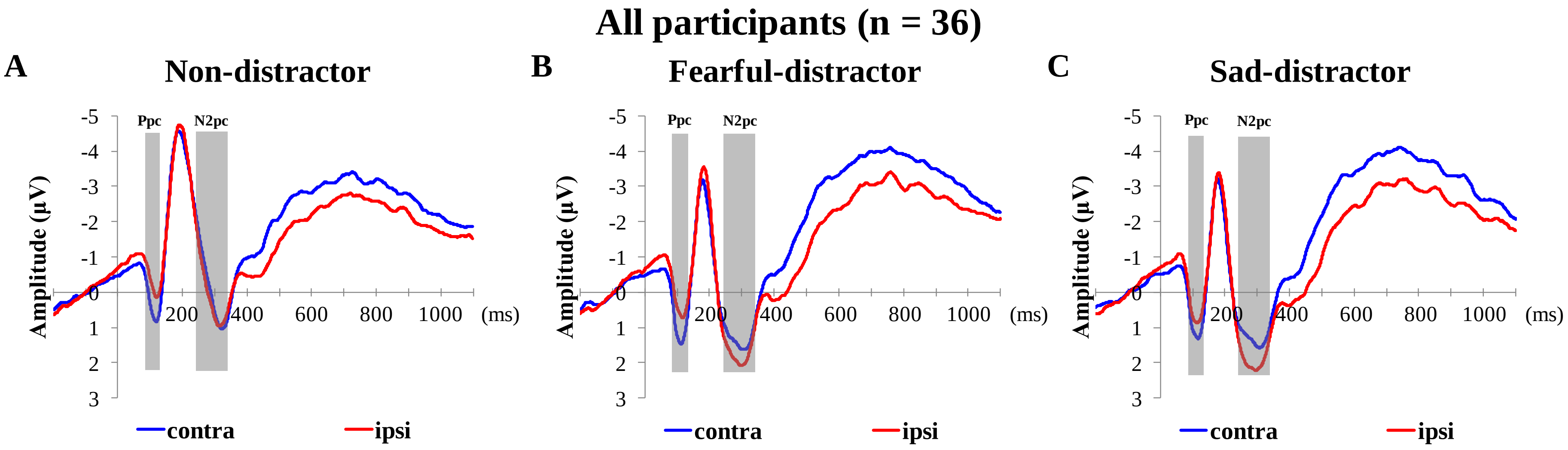

Fig. S2 Grand average ERPs recorded at PO7/PO8 elicited by memory arrays under (A) the non-distractor condition, (B) the fearful distractor condition, and (C) the sad distractor condition for all participants. Blue lines reflect the activity contralateral to the lateralized items, and red lines reflect the activity ipsilateral to the lateralized items. Gray shades indicate the analysis time window used to calculate the mean Ppc and N2pc amplitudes. The waveforms were time-locked to the onset of the memory array (y-axis on time zero). contra = contralateral, ipsi = ipsilateral

2. Exploratory analyses for Ppc and N2pc components

A visual inspection of the difference waveforms suggested that early contralateral positivity and contralateral negativity were elicited in all conditions and that their amplitudes in the sad distractor condition were at least numerically different from those in the non-distractor condition. Early contralateral positivity is referred to as the positivity posterior contralateral (Ppc) component (Corriveau et al., 2012), or the early distractor positivity (Pd) component (Feldmann-Wustefeld & Vogel, 2019). The Ppc (80–160 ms) can be elicited by targets or distractors (Corriveau et al., 2012), and it reflects the initial processing of stimuli (Fortier-Gauthier et al., 2012). The contralateral negativity is referred to as the N2pc component (Eimer, 1996; Hopf et al., 2000; Liu et al., 2016; Luck & Hillyard, 1994a, 1994b; Zhao et al., 2011) and has been widely used to investigate the deployment of attention (Liu et al., 2016; Luck & Hillyard, 1994a, 1994b; Zhao et al., 2011). A relatively common practice is to interpret the N2pc (180–320 ms) as an index of the deployment of covert spatial visual attention (Kiss et al., 2008) or of the onset of attentional engagement (Zivony et al., 2018). Therefore, based on our visual inspection and similar to previous studies using CDA (Corriveau et al., 2012; Feldmann-Wustefeld & Vogel, 2019), we conducted exploratory analyses to compare the Ppc and N2pc components under different conditions. The preprocessing and calculation of the amplitudes of the difference waveforms of the Ppc and N2pc components were conducted essentially as described for the CDA component.

Ppc (82–132 ms)

The Ppc was measured for each condition (non-dis, fearful-dis, and sad-dis) as the difference in the mean amplitude between the ipsilateral and contralateral waveforms at electrodes PO7/PO8 (Corriveau et al., 2012; Feldmann-Wustefeld & Vogel, 2019). As shown in Fig. S3A, we chose 82–132 ms after the onset of the memory array as the time window for the Ppc, which was defined as ±25 ms from the most positive peak between 80 ms and 160 ms in the difference waveform (the contralateral and

ipsilateral waveforms are provided in the Supplementary Materials, Fig. S2).

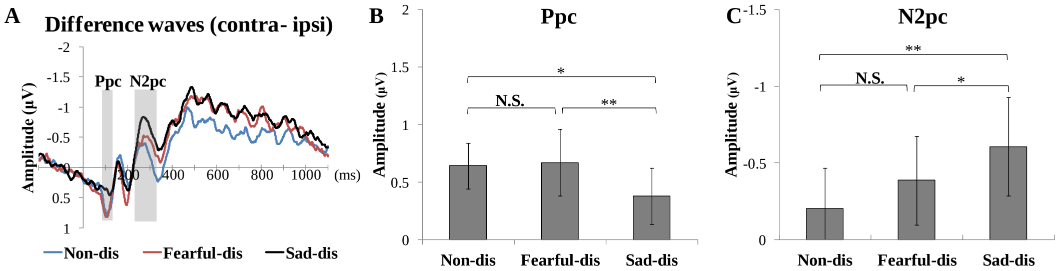

Fig. S3 Ppc and N2pc results. (A) Difference waves (contralateral waves minus ipsilateral waves) of the grand average ERPs at PO7/PO8 under different conditions elicited by memory arrays for all participants. Gray shades indicate the analysis time window used to calculate the mean Ppc and N2pc amplitudes. The waveforms are time-locked to the onset of the memory array (y-axis on time zero). (B) The results of the Ppc amplitude for all participants under each different condition. (C) The results of the N2pc amplitude for all participants under each condition. Bars show the mean amplitude values, and their error bars depict the 95% confidence interval of the mean. ** = p < 0.01; * = p < 0.05; N.S. = non - significant, i.e. p > 0.05. Non-dis = non-distractor condition, Fearful-dis = fearful distractor condition, Sad-dis = sad distractor condition, contra = contralateral, ipsi = ipsilateral.

The repeated measures ANOVA for the Ppc amplitude showed a significant main effect of condition (F(2,68) = 3.992, p = 0.023, η^2_p = 0.105). However, we found no significant main effect of group (F(1,34) = 2.417, p = 0.149, η^2_p = 0.060) or significant interaction of condition by group (F(2,68) = 0.425, p = 0.656, η^2_p = 0.012). Follow-up pairwise comparisons showed that the Ppc amplitude was significantly smaller in the sad distractor condition than in the fearful distractor condition (t(35) = 2.159, p = 0.017, Cohen's d = 0.368, BF_{10} = 2.698) and the non-distractor condition (t(35) = 2.952, p = 0.005, Cohen's d = 0.396, BF_{10} = 7.752). No significant difference was detected in the Ppc amplitude between the fearful distractor condition and the non-distractor condition (t(35) = 0.229, p = 0.820, Cohen's d = 0.050, BF_{10} = 0.183) (Fig. S3B). The Ppc amplitude was significantly larger than zero under the non-distractor condition (t(35) = 6.498, p < 0.001, Cohen's d = 1.083, BF_{10} > 10000), the fearful distractor condition (t(35) = 4.714, p < 0.001, Cohen's d = 0.786, BF_{10} =

603.00), and the sad distractor condition (t(35) = 3.154, p = 0.003, Cohen's d = 0.526, BF_{10} = 11.01). This indicated that the Ppc component was reliably observed in each condition.

We also investigated the relationship between the initial processing and memory storage of the distractors by examining the correlations between the Ppc difference scores and the CDA difference scores for the different distractors. The results showed that the Ppc difference scores were positively correlated with the CDA difference scores (r = 0.353, p = 0.035) in the sad distractor condition, whereas no correlation was found between the Ppc difference scores and the CDA difference scores for the fearful distractors (r = .283, p = .095).

Similar to the calculations shown in the main text, we also calculated the mean Ppc amplitude difference score (i.e., Ppc amplitude in fearful/sad distractor condition minus Ppc amplitude in the non-distractor condition). This allowed us to examine the correlations between the mean Ppc amplitude difference scores and amount of symptoms (i.e., BDI-II and DASS-A scores) to determine whether depressive symptoms (BDI-II scores) or anxiety symptoms (DASS-A scores) affected the initial processing of the distractors. The Ppc difference scores with a more positive value indicate a larger Ppc in the distractor condition than in the non-distractor condition, suggesting that larger initial processing is elicited by the distractors. No significant correlation was found between the BDI-II scores and the Ppc difference scores for fearful distractors (r = -0.041, p = 0.814) or sad distractors (r = - 0.106, p = 0.540). No significant correlation was found between the DASS-A scores and the Ppc difference scores for fearful distractors (r = -0.030, p = 0.862) or sad distractors (r = -0.062, p = 0.721).

N2pc (230–330 ms)

The N2pc was measured for each condition (non-dis, fearful-dis, and sad-dis) as the difference in the mean amplitude between the ipsilateral and contralateral waveforms recorded at the analyzed electrodes (PO7/PO8) (Feldmann-Wustefeld & Vogel, 2019; Luck & Hillyard, 1994a, 1994b) at 230–330 ms after the onset of the memory array

(Fig. S3A).

For the N2pc amplitude, repeated measures ANOVA showed a significant main effect of condition (F(2,68) = 5.002, p = 0.009, η^2_p = 0.128). However, no significant main effect was found for group (F(1,34) = 0.001, p = 0.978, η^2_p < 0.001) and no significant interaction was found for condition by group (F(2,68) = 0.020, p = 0.980, η^2_p = 0.001). Follow-up pairwise comparisons showed that, for all participants, the N2pc amplitude was significantly larger in the sad distractor condition than in the fearful distractor condition (t(35) = 2.159, p = 0.038, Cohen's d = 0.244, BF_{10} = 1.397) and in the non-distractor condition (t(35) = 2.952, p = 0.006, Cohen's d = 0.466, BF_{10} = 6.951). No significant difference was noted in the N2pc amplitude between the fearful distractor condition and the non-distractor condition (t(35) = 1.344, p = 0.188, Cohen's d = 0.223, BF_{10} = 0.409) (Fig. S3C). The N2pc amplitude was significantly larger than zero under the fearful distractor condition (t(35) = 2.699, p = 0.011, Cohen's d = 0.450, BF_{10} = 4.013) and the sad distractor condition (t(35) = 3.841, p < 0.001, Cohen's d = 0.648, BF_{10} = 59.116), but no significant difference was found between the N2pc amplitude and zero under the non-distractor condition (t(35) = 1.578, p = 0.124, Cohen's d = 0.263, BF_{10} = 0.553), suggesting that the N2pc component was reliably observed only in the fearful distractor condition and in the sad distractor condition.

We also investigated the relationship between attention allocation and memory storage of the distractors by examining the correlations between the N2pc difference scores and the CDA difference scores for the different distractors. The N2pc difference scores correlated positively with the CDA difference scores for the fearful distractors (r = 0.665, p < 0.001) and the sad distractors (r = 0.683, p < 0.001). We again calculated the mean N2pc amplitude difference score (i.e., N2pc amplitude in fearful/sad distractor condition minus N2pc amplitude in the non-distractor condition). The score was used to examine the correlations between the mean N2pc amplitude difference scores and amount of symptoms (i.e., BDI-II and DASS-A scores) to determine whether the depressive symptoms (BDI-II scores) or anxiety symptoms

(DASS-A scores) affected attentional processing toward distractors. The N2pc difference scores with more negative values indicate a larger N2pc in the distractor condition compared to the non-distractor condition, suggesting that participants allocate more attention to the cued hemifield. No significant correlation was found between the BDI-II scores and the N2pc difference scores for the fearful distractors ($r = -0.088$, $p = 0.610$) or the sad distractors ($r = -0.141$, $p = 0.413$). No significant correlation was found between the DASS-A scores and the N2pc difference scores for the fearful distractors ($r = -0.133$, $p = 0.440$) or the sad distractors ($r = -0.062$, $p = 0.721$).

Discussion

Very few studies have systematically analyzed the Ppc component (Corriveau et al., 2012; Feldmann-Wustefeld & Vogel, 2019; Jannati et al., 2013), and its functional meaning is not clear. The Ppc component may be related to the stimulus-driven P1 (75–125 ms), which is sometimes larger in areas contralateral to the cued array than in those ipsilateral to it. This lateralization of P1 has been attributed to low-level sensory processes (Luck & Hillyard, 1994a). In our study, the Ppc may be elicited by both the targets and the distractors in the pre-attentive phase. Our results indicated that the Ppc was smaller under the sad distractor condition than under the fearful face distractor condition or the non-distractor condition. The differences in the initial perceptional processing between sad distractors and fearful distractors may also be related to the allocation of more attention to the cued hemifield (enhanced N2pc) by the participants under the sad distractor condition than under the other conditions. We also found a positive correlation between the Ppc difference scores and the CDA difference scores for the sad distractor, suggesting that the change in initial perceptional processing is associated with difficulty in filtering sad face distractors. Future studies should investigate the functional meaning of the Ppc component in the processing of distractors.

In addition to examining CDA and Ppc, we conducted an exploratory analysis of the

N2pc component. Even if a well-documented negative bias toward sad faces exists in depression (e.g., Armstrong & Olatunji, 2012; Gotlib & Joormann, 2010), the CDA results did not indicate that task-irrelevant sad faces consumed working memory resources in the depressed group. The reason for the efficient filtering of sad faces in the depressed group is unclear. One possibility is that sad faces attracted the participants' attention, but they did not subsequently store those faces in VWM. The exploratory analysis showed that the N2pc was enlarged at the whole sample level in the sad distractor condition, but no group differences were found for the N2pc. Although our study is the first to investigate facial filtering in depression, our results suggest that the negative bias toward sad faces in depression (Armstrong & Olatunji, 2012) seems not to influence the maintenance phase of VWM. However, further studies are required to confirm this finding with a larger group of participants.

One notable result of the present study was a positive correlation between the N2pc difference scores and the CDA difference scores, which is in line with the findings reported by Salahub and Emrich (2020). Our results demonstrate an association between an increased likelihood of holding a facial distractor in VWM (as indicated by the CDA) and an increased allocation of attention to the face distractor (as indicated by the N2pc). Nevertheless, since the exploratory analyses of N2pc and Ppc were conducted post hoc (i.e., after observing the ERP waveforms), these findings should be interpreted with caution.

Reference

Armstrong, T., & Olatunji, B. O. (2012). Eye tracking of attention in the affective disorders: a meta-analytic review and synthesis. *Clin Psychol Rev, 32*(8), 704-723. doi: 10.1016/j.cpr.2012.09.004

Corriveau, I., Fortier-Gauthier, U., Pomerleau, V. J., McDonald, J. J., Dell'Acqua, R., & Jolicoeur, P. (2012). Electrophysiological evidence of multitasking impairment of attentional deployment reflects target-specific processing, not distractor inhibition. *International Journal of Psychophysiology, 86*(2), 152-159.

Eimer, M. (1996). The N2pc component as an indicator of attentional selectivity. *Electroencephalogr*

Clin Neurophysiol, 99(3), 225-234. doi: 10.1016/0013-4694(96)95711-9

Feldmann-Wustefeld, T., & Vogel, E. K. (2019). Neural Evidence for the Contribution of Active Suppression During Working Memory Filtering. *Cereb Cortex, 29*(2), 529-543. doi: 10.1093/cercor/bhx336

Fortier-Gauthier, U., Moffat, N., Dell'Acqua, R., McDonald, J. J., & Jolicœur, P. (2012). Contralateral cortical organisation of information in visual short-term memory: Evidence from lateralized brain activity during retrieval. *Neuropsychologia, 50*(8), 1748-1758.

Gotlib, I. H., & Joormann, J. (2010). Cognition and depression: current status and future directions. *Annu Rev Clin Psychol, 6*, 285-312. doi: 10.1146/annurev.clinpsy.121208.131305

Hopf, J. M., Luck, S. J., Girelli, M., Hagner, T., Mangun, G. R., Scheich, H., & Heinze, H. J. (2000). Neural sources of focused attention in visual search. *Cereb Cortex, 10*(12), 1233-1241. doi: 10.1093/cercor/10.12.1233

Jannati, A., Gaspar, J. M., & McDonald, J. J. (2013). Tracking target and distractor processing in fixed-feature visual search: evidence from human electrophysiology. *Journal of Experimental Psychology: Human Perception and Performance, 39*(6), 1713.

Kiss, M., Van Velzen, J., & Eimer, M. (2008). The N2pc component and its links to attention shifts and spatially selective visual processing. *Psychophysiology, 45*(2), 240-249. doi: 10.1111/j.1469-8986.2007.00611.x

Liu, Q., Lin, S., Zhao, G., & Roberson, D. (2016). The effect of modulating top-down attention deployment on the N2pc/PCN. *Biol Psychol, 117*, 187-193. doi: 10.1016/j.biopsycho.2016.04.004

Luck, S. J., & Hillyard, S. A. (1994a). Electrophysiological correlates of feature analysis during visual search. *Psychophysiology, 31*(3), 291-308. doi: 10.1111/j.1469-8986.1994.tb02218.x

Luck, S. J., & Hillyard, S. A. (1994b). Spatial filtering during visual search: evidence from human electrophysiology. *J Exp Psychol Hum Percept Perform, 20*(5), 1000-1014. doi: 10.1037//0096-1523.20.5.1000

Salahub, C., & Emrich, S. M. (2020). Fear not! Anxiety biases attentional enhancement of threat without impairing working memory filtering. *Cogn Affect Behav Neurosci, 20*(6), 1248-1260. doi: 10.3758/s13415-020-00831-3

Zhao, G., Liu, Q., Zhang, Y., Jiao, J., Zhang, Q., Sun, H., & Li, H. (2011). The amplitude of N2pc reflects the physical disparity between target item and distracters. *Neurosci Lett, 491*(1), 68-72. doi: 10.1016/j.neulet.2010.12.066

Zivony, A., Allon, A. S., Luria, R., & Lamy, D. (2018). Dissociating between the N2pc and attentional shifting: An attentional blink study. *Neuropsychologia, 121*, 153-163. doi: 10.1016/j.neuropsychologia.2018.11.003
